# A sociobiological origin of pregnancy failure in domestic dogs

**DOI:** 10.1038/srep22188

**Published:** 2016-02-26

**Authors:** Luděk Bartoš, Jitka Bartošová, Helena Chaloupková, Adam Dušek, Lenka Hradecká, Ivona Svobodová

**Affiliations:** 1 Department of Ethology, Institute of Animal Science, Přátelství 815, 10400 Praha Uhříněves, Czech Republic; 2 Department of Animal Science and Ethology, Faculty of Agrobiology, Food and Natural Resources, Czech University of Life Sciences Prague, Kamýcká 129, 16521 Praha 6-Suchdol Czech Republic.

## Abstract

Among domestic dog breeders it is common practice to transfer a domestic dog bitch out of her home environment for mating, bringing her back after the mating. If the home environment contains a male, who is not the father of the foetuses, there is a potential risk of future infanticide. We collected 621 records on mating of 249 healthy bitches of 11 breed-types. The highest proportion of successful pregnancies following mating occurred in bitches mated within their home pack and remaining there. Bitches mated elsewhere and then returned to a home containing at least one male had substantially lower incidence of maintained pregnancy in comparison with bitches mated by a home male. After returning home, housing affected strongly the frequency of pregnancy success. Bitches mated elsewhere but released into a home pack containing a home male were four times more likely to maintain pregnancy than bitches which were housed individually after returning home. Suppression of pregnancy in situations where a bitch is unable to confuse a home male about parentage may be seen as an adaptation to avoid any seemingly unavoidable future loss of her progeny to infanticide after birth and thus to save energy.

Multi-male mating is common among nearly 90% of 40 carnivore species in which it is known that offspring may be vulnerable to infanticide^1^. The most credible explanation is that multi-male mating confuses paternity, thereby deterring males from potential infanticide^1^,^2^. Infanticide as a male’s reproductive strategy has been rarely reported in dogs^3^; Pal 2012 personal communications. However, in free ranging domestic dog where pup mortality is enormous ranging from 5–35%^4^ it is usually difficult to get any information about the causes of mortality and there is some indication that part of this might be attributed to infanticide e.g.^3^,^5^,^6^,^7^. It seems unlikely that such adaptations have been lost through domestication^8^,^9^,^10^,^11^.

The relatively rare incidence of male infanticide in dogs may be a result of efficient counter-strategies of females. Besides multi-male mating^1^,^2^,^12^ designed to cause confusion about paternity, an actual blocking of pregnancy in response to the proximity of strange males (“Bruce effect”) has been suggested as another potential counter-strategy to the risk of infanticide^2^,^13^. This was first observed in the laboratory house mouse *Mus musculus*^14^,^15^ where exposure to the urinary scent of an unfamiliar male within a limited time after mating appeared to inhibit implantation of the fertilized ovum^16^,^17^. Although the “Bruce effect” was originally investigated in a domesticated house mouse, it has since been reported also for wild rodents, albeit under captive conditions^18^,^19^,^20^,^21^,^22^,^23^,^24^.

A different mechanism has been reported by Bartoš *et al*.^25^ in the domestic horse *Equus caballus*. In this case, mares were already known to be pregnant before they met and/or could meet a male who was not the father of the foetus and thus pregnancy disruption necessarily involves active termination of pregnancy. Mares which returned home from away-matings^25^ or were artificially inseminated^26^ and were housed in enclosures together with a home male showed repeated sexual activity. Pregnancy disruption of the mares was seven to eight times more likely when the mare had no male company in her enclosure but when home stallions or geldings were present in an adjacent enclosure than when the mare was sharing the enclosure with home male(s). Such results suggest that when sharing the same enclosure with male or males, a mare manipulated the male’s paternity assessment by promiscuous mating as a counter-strategy against potential future infanticide. If she has no chance to do that, she terminated her pregnancy. We interpreted this effect as a female counter-strategy to possible male infanticide^25^,^26^. Disruption of pregnancy, post-implantation, has also been reported in several species of free ranging primates^27^,^28^,^29^,^30^,^31^,^32^. All this evidence suggests that the active disruption of pregnancy in situations where there is significant high risk of subsequent infanticide of progeny carried full term is adaptive rather than unfortunate.

The aim of this paper is to contribute further to a discussion about the relevance of theoretical frameworks derived from behavioural ecology and sociobiology in solving problems in domestic animals. The main differences between ancestral and domestic species are the environmental constraints imposed by natural and domestic environments, the different selection pressures, present and past, acting on wild and domesticated animals and the effects (deliberate or otherwise) of centuries of selective breeding within the domesticated form. However, there is little evidence suggesting that domestication has resulted in actual loss of behaviours from any species repertoire, that addition of new behaviours has occurred or that domestic animals are incapable of making optimal decisions in their environment^33^. Here we test the presumption that unlike for example adaptation to a human-controlled diet^34^, at least some behavioural strategies and responses developed in its wild ancestors over evolutionary time remain present in the domestic dog even after many generations of domestication and selective breeding.

Failure to conceive, or the subsequent failure of pregnancy in female domestic dogs *Canis lupus familiaris* may result in significant breeding and financial losses; in consequence there has been considerable research effort to attempt to determine likely causes. However, reasons for pregnancy failure are often complex and multifactorial. Improper timing of mating is one of the most common reasons why bitches may fail to conceive^35^,^36^. Viruses or other disease agents, together with non-infectious factors such as housing and social conditions are among the causes most commonly considered to be responsible for subsequent loss of a litter in pregnant bitches^25^,^26^. In discussions of unexplained reproductive failure in humans, stress has frequently been cited as a factor^37^, but, with few exceptions, e.g.^38^, the veterinary literature has revealed almost no evidence of effects of stress on reproduction in dogs.

Infanticide may not only be a risk in relation to non-paternal males. Females of various species, including canids, may improve their own reproduction by suppressing reproduction of their subordinate conspecifics reviewed by^39^,^40^. In canids such as the wolf *Canis lupus*, there may be a significant effect of reproductive suppression of a low-ranking female as a result of the presence of other more dominant females^41^,^42^. Where pups are actually born to subordinate individuals these may subsequently be killed by other females, or even by the mother, as well as by non-paternal males^3^. In early studies on free-ranging dogs, group splitting during denning was explained as an adaptive strategy as a way for subordinate females to avoid the threats of aggression by the dominant female^3^. In studying reproduction in the domestic dog, the potential influence of other females in the group has to be considered as well as the influence of pack males.

As with domestic horses^25^, it is common practice among the domestic dog breeders to transfer a bitch out of her home environment for mating and bringing her back after the mating. If the home environment contains a male, who is not the father of the foetuses, or other more dominant females, there may be a potential risk for future infanticide. In this study we investigated if female dogs show an adaptive strategy of pregnancy termination to reduce such a risk. We predicted that (i) the lowest incidence of pregnancy failure following mating will occur in bitches mated within their home pack; (ii) pregnancy failure will occur more frequently in bitches mated elsewhere and brought back to a home environment that contains a home male or males; (iii) bitches mated elsewhere which were individually housed and therefore separated from a home male after returning home would display pregnancy failure more frequently then bitches mated elsewhere but sharing the space with a home male (because through lack of access to the home male there were unable to confuse the male about paternity of the pregnancy). We also predicted that (iv) pregnancy failure of bitches mated elsewhere but released into a home pack containing a home male should not differ from releasing into a home pack with no male present, because if the bitch is able to mate freely with the home male, she can satisfactorily confuse him about paternity of the litter thus removing risk of subsequent infanticide. Finally (v) we investigated the extent to which pregnancy failure might be influenced by the number of other bitches.

## Results

We collected 621 records on mating 249 healthy bitches of 11 breed types (see Table 1) from 103 breeders. Within this sample we have documented one case of infanticide by the male and 12 cases of female infanticide. This may be underestimated, however, because respondents apparently did not pay much attention to these questions answering predominantly “I do not know” (Table 2).

Pregnancy tests are not common among domestic or commercial breeders and in our analyses below, we defined a “pregnancy failure” as a failure to produce viable pups over the period of 65 days after observed mating finalised by a coital tie or lock^43^.

**Figure 1:**
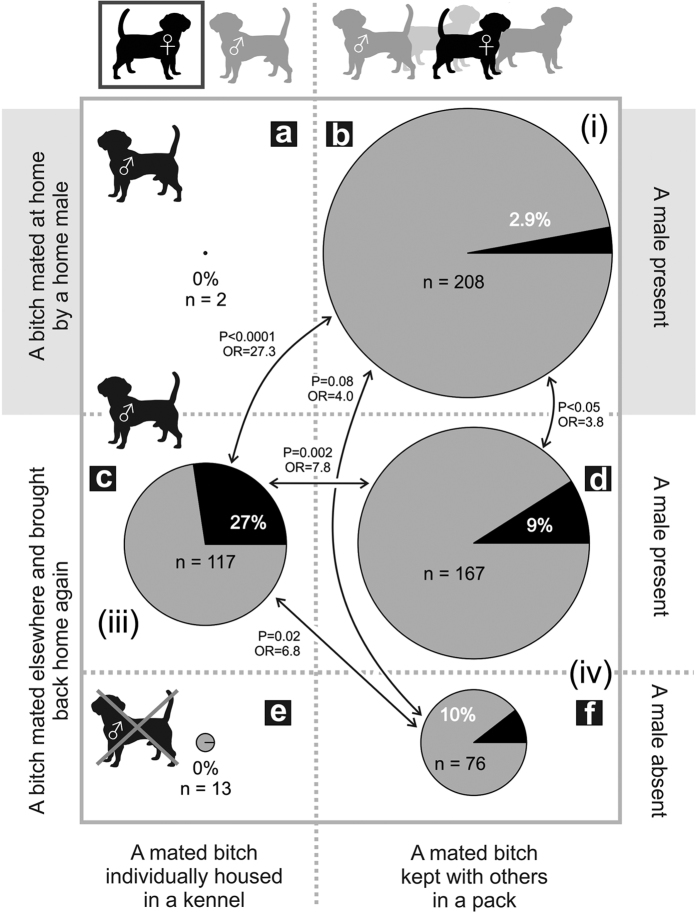
Proportion of pregnancy failure for bitches after mating. Legend: Proportion of pregnancy failures for bitches mated elsewhere and brought back again (top and middle pairs of pies) or bitches mated at home by a home male (bottom pair of pies), according to whether the mated bitch was individually housed and therefore separated from other dogs (left column of pies) or kept with others in a group (right column of pies), and according to if a home male was present (top and bottom pairs of pies) or absent (middle pair of pies). The proportion of pregnancy failure is indicated by black slice of the pie and is expressed by percent of pregnancy failures of all mating within the combination reflected by the pie. N represents the number of cases for the pie which is also reflected by the size of the pie. OR is odds ratio. Roman numerals (i, iii, and iv) indicate the predictions advanced. (The figure was drawn by LB).

**Figure 2:**
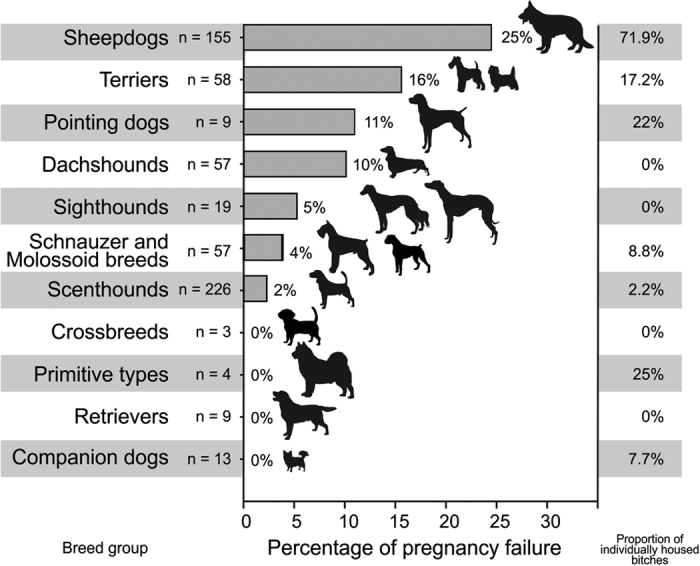
Proportion of pregnancy failure for bitches after mating. Legend: Proportion of pregnancy failures (horizontal bars with the percent number), number of records and proportion of individually housed bitches for each of the breed-types involved in the GLMM. (The figure was drawn by LB).

**Figure 3:**
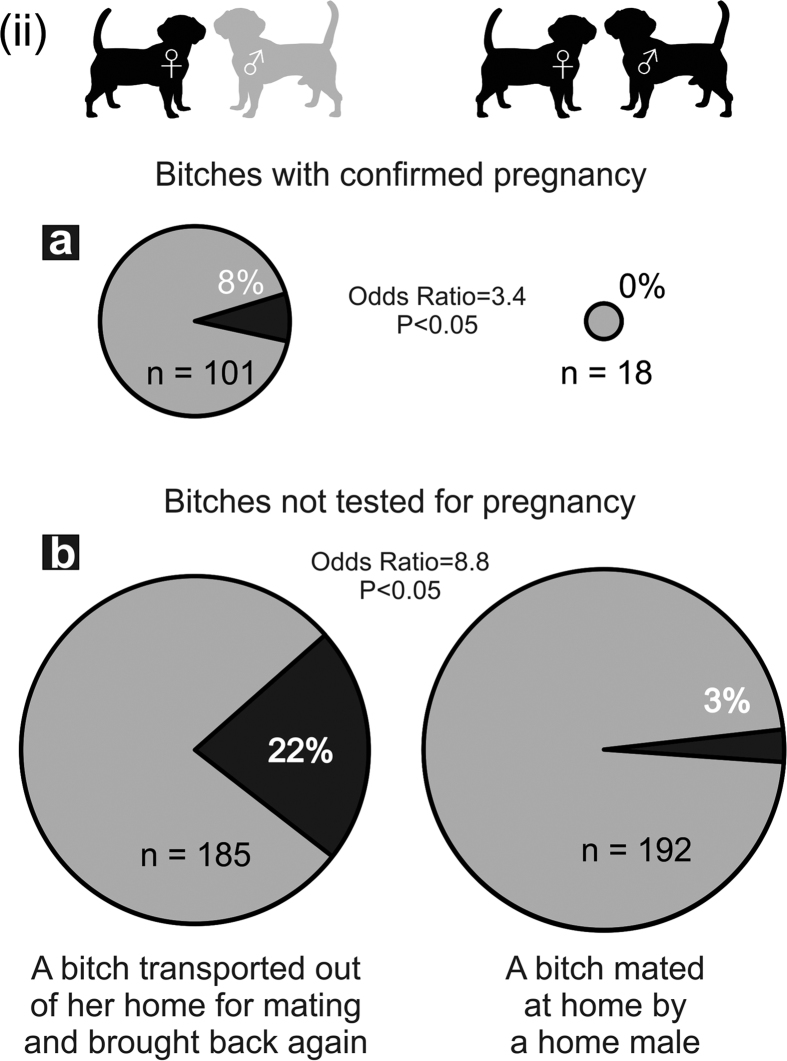
Comparison of proportion of pregnancy failure after mating between bitches where pregnancy had actually been confirmed by ultrasound and bitches with no pregnancy testing. Legend: Proportion of pregnancy failure according to whether a bitch was transported for mating with a new male elsewhere and brought back home in an environment containing a home male in comparison with that of bitches mated and then kept at home. Upper row of pies shows proportions of bitches with confirmed pregnancy, lower row shows proportions for bitches not tested for pregnancy. (The pie size reflects the number of cases and Roman numeral ii the prediction advanced). (The figure was drawn by LB).

As predicted (i), the lowest incidence of pregnancy failure occurred in bitches mated and then kept in the same group containing a home male (2.86% failures out of 210 records), as estimated by odds ratio, some 4 to nearly 27 times lower than levels reported under other conditions, Fig. 1b, Table 3A).

(ii) Bitches mated elsewhere and then returned to a home pack containing at least one male showed 16.78% failures (out of 286 records), a failure rate approximately 3 times higher in comparison to bitches mated by a home male (Odds ratio = 3.21, Table 3B). Frequency of pregnancy loss was also affected by breed type (Table 3B, Fig. 2). Sheepdogs were more prone to pregnancy failure while sighthounds, scenthounds, schnauzer and molossoid breeds, crossbreeds, primitive types, retrievers and companion dogs were less prone to reproductive failure. The proportion of bitches showing pregnancy failure and the proportion of bitches which were individually housed (Fig. 3) were positively correlated (Pearson correlation r = 0.74, P < 0.02) conforming to our prediction that bitches mated elsewhere which were separated from a home male and/or females after returning home would display pregnancy failure more frequently then bitches mated elsewhere but sharing the space with a home male.

Records where pregnancy was subsequently confirmed by ultrasonic diagnosis were relatively few and did not allow us to test all the full range of possible combinations beyond a simple comparison of odds ratios between the bitches mated elsewhere and then returned to their home containing at least one male, and bitches mated and then kept in the same group containing a home male. Eight bitches mated elsewhere and then returned to their home showed pregnancy disruption while all bitches mated by a home male maintained pregnancy (Fig. 3a, Χ^2^_(1)_ = 1.52, P = 0.22). Although the difference between pregnancy failures of bitches mated elsewhere and then returned home and those mated at home did not reach formal levels of significance, the relative proportion of pregnancy failures of bitches in the different categories was similar in the sample with no pregnancy testing data available (Fig. 3b, Χ^2^_(1)_ = 31.21, P < 0.0001) and the Breslow-Day statistic did not contradict the assumption of homogeneous odds ratios in bitches ultrasonically confirmed pregnant and those not tested (Χ^2^_(1)_ = 0.182, P = 0.67).

(iii) The probability of pregnancy failure strongly depended on where the bitch was mated, in association with presence of a home male, in interaction with housing of the bitch (Table 3A, Fig. 1). (Eight bitches for sure disrupted their pregnancy because their pregnancy was confirmed). Pregnancy failures were recorded for almost one third of bitches mated away from home and housed individually after return home (with thus no access to the home male; Fig. 1c). This loss rate was over seven times higher than when a bitch returned into a home pack with a male and was allowed to mix freely with other pack members (Fig. 1d). Bitches mated elsewhere and individually housed after returning home with a home male absent were too rare to be reasonably compared (Fig. 1e).

(iv) In agreement with our predictions, the probability of pregnancy failure in bitches mated elsewhere and released into a home pack containing a home male (Fig. 1d) was equal to that after release into a home pack with no male present (Fig. 1f). However, bitches mated elsewhere and released into a home pack (with or without a home male: Fig. 1d,f), still had four times higher percentage of pregnancy failure than bitches mated at home and subsequently maintained within the home group (Fig. 1b).

**Figure 4:**
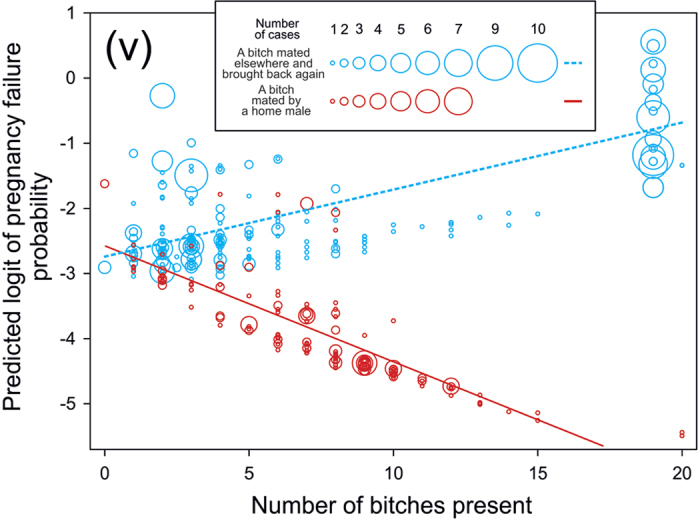
Proportion of pregnancy failure for bitches after mating. Legend: Predicted probability of pregnancy failure plotted against the number of bitches present in the home environment according to whether a bitch was mated elsewhere by a new male and brought back home again or whether a bitch was mated by a home male. Roman numeral (v) indicates the prediction advanced. (The figure was drawn by LB).

(v) Probability of pregnancy failure was affected by the number of bitches present in the home environment but the effect differed in relation to where a bitch was mated (Table 3C, Fig. 4). When a bitch was mated elsewhere and brought back, the probability of pregnancy failure increased with increasing number of bitches present (Solution for fixed effects, slope = 0.08, t = 2.50, P = 0.012). When a bitch was mated by a home male, there was, by contrast, some indication of a decrease in the probability of pregnancy failure with increasing number of bitches present, although this was not significant (slope =−0.14, t =−1.62, NS). Inspection of the resulting data showed unequal distribution of the number of bitches present. To check if the result was affected by the outlying values only, we removed outlying values (i.e., values with the number of bitches present 19 and 20, see Fig. 4) and ran the GLMM again.

Dependence on the number of other females disappeared for bitches mated elsewhere (slope =−0.17, t =−1.51, NS), while the one for bitches mated by a home male showed significant decrease (slope =−0.27, t =−2.81, P = 0.005).

In the next step, we included in the model the interaction between where a bitch was mated and where she was housed (Table 3E). The probability of pregnancy failure increased with increasing number of bitches when a bitch was housed individually (slope = 0.030, t = 2.62, P = 0.009), while when she was kept in a group the probability of pregnancy failure decreased with increasing number of bitches (slope = −0.18, t = −2.28, P = 0.02). Removing outlying values from this model also, the difference between bitches housed individually and those maintained within a group remained statistically significant (Table 3 F); however, only in grouped bitches was there a decreasing trend of the probability of pregnancy failure with the increasing number of bitches (Solution for fixed effects, slope = −0.25, t = −2.86, P = 0.005).

## Discussion

In agreement with our first prediction (i) the lowest incidence of pregnancy failure occurred in bitches mated and then kept in the same group containing a home male. (ii) Bitches mated elsewhere and then returned to a home environment containing at least one male had substantially higher incidence of the pregnancy failure than bitches mated by a home male. Taking a bitch for mating elsewhere unquestionably increases the chance for missing the optimum timing for conception which is usually reported as the most common reason why breeding fails, e.g.^35^,^44^. However, improper timing does not seem to be the major factor in this study, because, rates of successful pregnancy were not related to the number of matings which occurred within any given oestrus (Table 1); in addition, there was a highly significant effect of social environment on the probability of maintaining a pregnancy after returning home from mating elsewhere (Fig. 1). Bitches mated elsewhere which were separated from a home male after returning home showed the highest incidence of pregnancy failure. By comparison, bitches mated elsewhere but released into a home pack containing a home male showed nearly four times lower incidence of pregnancy failure when compared to bitches separated in a kennel after mating elsewhere (prediction iii).

Pregnancy failure of bitches mated elsewhere but released into a home pack containing a home male did not differ from that of bitches released into a home pack with no male present.

These results can most simply be explained as a direct result of a lack of opportunity for separately-housed females to confuse home male(s) about parentage. In various mammalian species, it is reported that pregnant females copulate with foreign males they encounter to confuse paternity^2^,^25^,^45^.

The benefit of this counter-strategy would be that males that have copulated with a given female are generally inhibited from killing young for the time period in which their offspring would be vulnerable to infanticide^13^,^46^,^47^,^48^.

Finally we have shown that the number of other adult females within the home pack appears to affect the probability of a mated bitch maintaining pregnancy (v), albeit in a rather complex manner. Aggressive agonistic encounters between adult group members of the free-ranging dogs are common, the level of aggression being higher in adult females than in adult males^49^. Particularly if pregnancy failure may be related to some extent to stress^41^,^42^,^50^,^51^,^52^, the presence and number of other bitches in the home environment might be expected to have some effect on the probability of such failure to maintain pregnancy. We found that after returning a bitch from mating elsewhere, the probability of pregnancy failure generally increased with the number of other bitches present. However, biased distribution of the records cast doubts about the wider validity of this initial conclusion; when records from the largest breeding station were removed, the increasing trend for bitches mated elsewhere disappeared, although the differences between bitches mated elsewhere and at home remained significant. This is clearly one interesting area where more research is needed.

We have found statistical differences between dog breeds in rates of pregnancy failure or failure to conceive. There is no doubt that dog breeds differ genetically, e.g.^53^,^54^. However, breed-type itself was not strong enough to reach significance once effects of the social environment of a returning bitch was incorporated into a GLMM (whether or not the bitch was individually or socially housed). This together with a high correlation between the proportion of pregnancy failures and the proportion of individually housed bitches suggests that social factors have a stronger impact on the probability of pregnancy failure than any genetic factors. We argue that transferring a domestic dog bitch for mating out of her home and bringing her back to the home environment containing a male, who is not father of the foetuses, induces a potential danger for future infanticide. As a counter-strategy against possible infanticide, pregnancy failure may follow. Highly unequal contribution of dog breeds and housing conditions, usually typical for a given breed in our study indicates a need of further research focussed on different dog breeds and their management.

At this stage our data could not resolve whether pregnancy failure was predominantly caused either by failure to conceive as in classical Bruce effect with rodents e.g.^14^,^15^,^18^,^19^,^20^,^24^, or by pregnancy disruption as in horses^25^,^26^. However, at least some of the bitches in this study successfully conceived (as apparent from ultrasonic scans) during the pre-implantation period generally characterized by increased vulnerability of early pregnancy to stressors^55^ but then subsequently disrupted that pregnancy. Proportions of pregnancy failure termination resulting from mating elsewhere or at home were similar for results from bitches with confirmed pregnancy or from bitches with untested pregnancy. This suggests that in terms of counter-strategy against infanticide domestic dogs might be closer to domestic horses^25^,^26^ (in disrupting at least a proportion of pregnancies post-implantation), rather than to various rodents^18^,^19^,^20^,^24^,^56^, etc., where response mechanisms tend to act primarily at a pre-implantation stage. This needs more data and is definitely an area for future research.

By providing evidence that pregnancy failure in domestic dog may derive from an evolutionary counter-strategy against infanticide, our results support the concept that a number of strategies and responses developed in its wild ancestors over evolutionary time remain present even after many generations of domestication. Practical implications of these findings are that to increase probability of successful pregnancy, bitches mated elsewhere are better kept together with other home dogs after their return home rather than separated and housed individually.

## Methods

In this study, we analysed data collected by questionnaire (Table 1) distributed through the internet to domestic dog breeders in the Czech Republic. Data were returned in this survey for bitches that were kept individually, or with one or more bitches or males present, and where returning bitches were kept together with other dogs or physically separated from other dogs but which she could see, hear or smell.

In the domestic dog, taking a bitch for mating elsewhere is usually a matter of a few hours or a few days absence from the home kennel. Pregnancy tests are not common and in our analyses therefore, we defined a “pregnancy failure” as a failure to produce viable pups over the period of 65 days after observed mating finalised by a coital tie or lock^43^.

Breeders reported confirmed pregnancy by testing in 303 pregnancies. However, because of problematic accuracy of some of the testing methods, as “confirmed pregnancy” we only accepted those cases obtained by ultrasonic diagnostic (n = 119) applied between 26 and more days post coitum.

### Ethics Statement

Data were collected in accordance with the Guide for the Care and Use of Animals of the Czech University of Life Sciences Prague and all experimental protocols were approved by the Faculty of Agrobiology, Food and Natural Resources licensing committee (Permit number: MŠMT 26663/2010-30, 7/2010).

### Statistics

Data were analysed using a Generalized Linear Mixed Models (GLMM, PROC GLIMMIX for binary distribution; SAS version 9.4) with binary dependent variable “pregnancy failure” (the bitch got pregnant after the mating/the bitch failed to get pregnant) modelling the probability that the bitch failed to get pregnant. The link function was logit and all error terms were binomial in the GLMM. The goodness of fit of each model (homoscedasticity, normality of errors and independence) was checked by visual inspection of residuals using plots = pearsonpanel and testing residuals for normality by Kolmogorov-Smirnov test. Predictors and fixed effects employed within both models are summarised in Table 1. We constructed the models by entering first that factor expected to have an effect on the bitch’s probability of blocking reproduction and then revising the model with addition of the Breed type (Table 1) to ensure that the breed-type was not contributing to the probability of pregnancy failure. When an effect of Breed type could be excluded because of non-significance, we added others of the factors listed in Table 1 which might potentially also influence the result. Any factors which did not add to significance (P > 0.05) were dropped from the model and will not be mentioned any further. When fitting the best GLMM model, we followed the “Fit Statistics” table providing several information criteria: AIC^57^, AICC^58^,^59^, and BIC^60^, all in smaller-is-better form.

We constructed first a complex GLMM to answer three predictions simultaneously, (i), (iii), and (iv), which tested the probability of pregnancy failure in relation to a) where the bitch was mated and b) the presence or absence of a home male(s) after an away mated bitch returned to the home group, in interaction with c) housing of the bitch (i.e., if she was individually housed/kept in a group). These three factors entered the model as a three-way interaction. The analysis was first performed with individual bitches nested within the ID of the breeder as a random effect. Because the procedure did not converge, as recommended by Kiernan *et al*.^61^ we used a different method (Method = QUAD) and SUBJECT = bitches nested within the ID of the breeder option in the RANDOM statement required for the method = QUAD.

(ii) To test the second prediction (that a bitch mated away from home and returned to a home environment with a resident male was less likely to maintain pregnancy that a bitch mated and maintained within the home group) we focused on fixed effects of where a bitch was mated (bitches transported elsewhere for mating by a “new male” or at home with a home male) and a breed-type (11 levels). For this we used only the data when there was at least one “home male” present. To account for the use of repeated measures on the same individuals in this model, the analysis was performed using mixed model with an individual bitch as a random effect.

(v) In the last model we tested the effect of the number of other bitches within the home environment nested within the fact if a home male was present or not for the bitches mated elsewhere and returned home.

To compare whether the chance of a certain event to occur differed in any two groups, we computed the odds ratio^62^. In PROC GLIMMIX and PROC FREQ the odds ratio is computed as the exponentiation of a difference on the logit scale. An odds ratio greater than one implies that the event is more likely to occur in the first group, whilst an odds ratio less than one implies that the event is more likely to occur in the second group. To compare relationship between proportion of pregnancy failure and proportion of individually housed bitches, Pearson correlation was applied (PROC CORR). The tests were two-tailed.

For detecting possible incongruity in the incidence of pregnancy failure particular circumstances between bitches in which pregnancy was confirmed via ultrasonic diagnostics and those who were not tested, we performed stratified analysis of contingency table and tested the homogeneity of odds ratios in a set of tables using a Breslow-Day statistic^63^ (PROC FREQ).

## Additional Information

**How to cite this article**: Bartoš, L. *et al*. A sociobiological origin of pregnancy failure in domestic dogs. *Sci. Rep*. **6**, 22188; doi: 10.1038/srep22188 (2016).

## Figures and Tables

**Table 1:** Questionnaire on reproduction of the bitches involved and conditions under which the bitches were living.

Characteristic	Range or categories
Year of the bitch’s birth	1986–2004
Age of the bitch	1–28 years
Breed type	Different breeds of dogs summarized into 11 breed types*
Number of puppies the bitch had delivered	0–57
If the bitch was transported during her pregnancy	Yes (n = 336)/No (n = 232)
How was the bitch housed	Individually (n = 133)/In a group (n = 451)
After the bitch returned from mating elsewhere, a home male was present	Yes (n = 497)/No (n = 89)
How many times was the bitch mated or artificially inseminated within the oestrus	1–6
The bitch transported elsewhere for mating	Yes (n = 375)/No (210)
Method of breeding	Natural mating (n = 571)/Artificial insemination (n = 15)
Date of delivery	From 22 April 2005 to 1 July 2009
Number of adult bitches kept within the facility regardless of the housing system	1–20
Number of adult home males kept within the facility regardless of the housing system	0–9
Where were the adult bitches kept within the facility (even if the other bitches were kept out of the kennel, the focal bitch could see and/or hear and smell them)	Same kennel as the pregnant bitch (n = 451)/out of the kennel (n = 133)

*Sheepdogs (Australian shepherd n = 1, Bearded collie n = 2, Beauceron n = 1, Belgian shepherd n = 26, Border collie n = 3, Briard n = 5, German shepherd n = 114, Rough collie n = 2), Terriers (American pitbull Terrier n = 2, Bedlington terrier n = 1, Border terrier n = 32, Bullterrier n = 5, Cairn terrier n = 8, Fox terrier n = 1, Manchester terrier n = 1, West highland white terrier n = 8), Pointing dogs (Bohemian wire-haired pointing griffon n = 1, German pointer n = 2, Hungarian vizsla n = 2, Irish setter n = 3, Weimaraner n = 1), Dachshounds (n = 57), Sighthounds (Afghan hound n = 1, Borzoi n = 2, Saluki n = 15), Schnauzer and Molossoid breeds (Argentine dog n = 1, Boxer n = 1, Bulldog n = 1, Caucasian shepherd dog n = 1, Fila Brazileiro n = 6, Great dane n = 3, Mastiff n = 1, Rottweiler n = 1, Schnauzer n = 37), Scenthounds (Beagle n = 202, Dalmatian n = 2, Rhodesian ridgeback n = 8), Crossbreeds (n = 3), Primitive types (Alaskan malamute n = 1, German spitz n = 1, Siberian husky n = 2), Retrievers (Portuguese water dog n = 1, Retriever n = 6), Companion dogs (Bolognese n = 1, Chihuahua n = 3, Chinese crested dog n = 4, Poodle n = 1, Prague ratter n = 2, Yorkshire terrier n = 1).

**Table 2:** Additional information on a bitch mated elsewhere after returning to home environment.

	Yes	No	I do not know
Mated bitch solicited home male	1	—	299
A home male attacked puppies of the elsewhere mated female	1	2	297
A home male attacked the mated bitch shortly after her return	1	—	299
Other bitch attacked and killed puppies of the elsewhere mated female	12	1	287

**Table 3:** Results of GLMMs for the five advanced predictions with dependent variable “pregnancy failure”.

	Fixed effect	Num DF	Den DF	F Value	Probability
A	Prediction (i), (iii), and (iv)				
	Where a bitch was mated (Presence of a home male * Housing of the bitch)	5	576	4.92	0.0007
B	Predictions (ii)				
	Where a bitch was mated	1	484	5.18	0.023
	Breed type	9	484	2.10	0.028
C	Prediction (v) first step				
	Number of bitches present (Where was a bitch mated)	2	320	6.40	0.0019
D	Prediction (v) first step—with outlining values removed				
	Number of bitches present (Where was a bitch mated)	2	259	3.95	0.0205
E	Prediction (v) second step				
	Where a bitch was mated (Housing of the bitch)	4	498	3.33	0.010
F	Prediction (v) second step—with outlining values removed				
	Where a bitch was mated (Housing of the bitch)	2	258	4.15	0.017
